# Sagittal Spinal and Pelvic Postures of Highly-Trained Young Canoeists

**DOI:** 10.2478/v10078-011-0038-5

**Published:** 2011-10-04

**Authors:** Pedro A. López-Miñarro, José M. Muyor, Fernando Alacid

**Affiliations:** 1 Department of Physical Education. University of Murcia; 2 Department of Physical Education. University of Almería; 3 Department of Physical Activity and Sports. University of Murcia

**Keywords:** thoracic, lumbar, pelvic, spine, posture, canoeists

## Abstract

The objective of this study was to determine the sagittal spinal curvatures and pelvic position in standing and kneeling in the canoe in young canoeists. Forty-four young highly-trained canoeists (mean age: 15.11 ± 0.61 years) were recruited. Thoracic and lumbar curvatures and pelvic inclination were evaluated with a Spinal Mouse system in standing position and in the base position (kneeling on one knee in the canoe) and catch phase of the stroke. The mean thoracic kyphosis, lumbar lordosis and pelvic inclination in standing were 44.66 ± 8.80º, −30.34 ± 8.31º, and 14.20 ± 7.32º, respectively. In the canoe, the thoracic, lumbar and pelvic angles were 39.66 ± 9.52º, −24.32 ± 6.79º, and 15.18 ± 4.34º, respectively, for the base position (p<0.001 with respect to standing, except for pelvic inclination), and 28.93 ± 10.45º, −13.45 ± 10.60º, and 37.61 ± 6.27º, respectively, for the catch phase of the stroke (p<0.001 with respect to standing and base position). A higher percentage of hyperkyphotic postures in standing than in the canoe was found, while thoracic hypokyphosis increased in the catch phase of the stroke. In regards to the lumbar curve, the percentage of hypolordosis postures in the base position was higher than when standing. Lumbar kyphotic postures were detected in the catch phase of the stroke. In conclusion, the standing thoracic hyperkyphosis in young canoeists may be related to factors other than the posture and movement in the canoe. The canoeists adopted a lumbar flexed posture at the catch phase of the stroke, although this position may not affect the sagittal configuration of lumbar spine in standing. Postural training should be included in the training program of canoeists to improve the thoracic posture in the standing position.

## Introduction

Sagittal spinal curvatures may adapt gradually to one’s sport if training is conducted intensively for long periods. Sagittal spinal curvatures are geometric parameters which influence mechanical properties and load balance of the intervertebral disc (Wilke et al., 2001; McGill, 2002; Polga et al., 2004; Keller et al., 2005). Exposure to years of intense athletic training may increase the risk of developing spinal disorders in certain sport disciplines (Wojtys et al., 2000; Muyor et al., 2011a,b).

Previous studies have found a relationship between sport training and changes in sagittal spinal curvatures. Athletes participating in rigorous sports had a higher risk of thoracic hyperkyphotic than sedentary people (Wojtys et al., 2000). Sports with a predominance of bending postures have been related to an increased thoracic kyphosis in standing. Rajabi et al. (2008) found that the thoracic kyphosis was higher in freestyle than in Greco-Roman wrestlers. The freestyle routinely puts the spine in a more flexed position while the Greco-Roman style uses the spine in an almost erect position. Alricsson and Werner (2006) reported an increased thoracic kyphosis in adolescent elite cross-country skiers after a period of 5 years of intensive training, but no change for the lumbar lordosis. Other studies found greater thoracic and lumbar flexion in paddlers with respect to sedentary subjects (López-Miñarro et al., 2010a) and runners (López-Miñarro et al., 2009) when the sit-and-reach test was performed. Förster et al. (2009) demonstrated that spinal curvatures of climbers were characterized by increased thoracic kyphosis and lumbar lordosis. Furthermore, the climbing ability level was strongly correlated to the postural adaptations. Wojtys et al. (2000) also demonstrated that larger angles of thoracic kyphosis and lumbar lordosis were associated with a greater cumulative training time in a growing spine.

Some sport disciplines with an important spinal implication have not been analyzed. The canoeing discipline of sprint paddling is basically a repetitive movement of the upper segments and trunk for long periods. Canoeing is characterized by kneeling on one knee in a canoe and performing dynamic strokes on one side only. The stroke requires significant flexion and rotation of the trunk and a slight lateral inclination. As canoeists spend a large amount of time training in their boats to elicit a physiological training effect, this may influence their thoracic and lumbar spinal curvatures in standing.

López-Miñarro et al. (2008) found a high frequency of thoracic hyperkyphosis in canoeists and justified this fact as an adaptation to the trunk position during stroke. Alterations in spinal curvatures may potentially influence the development of low-back pain (Harrison et al., 2005; Smith et al., 2008), which is a common injury among canoeists (Kameyama et al., 1993). However, no studies have analyzed the specific paddling posture. For this reason, the objectives of this study were: 1) to describe the thoracic and lumbar posture and pelvic inclination while standing in young canoeists; and 2) to compare the spinal posture and pelvic inclination between standing and paddling posture.

## Material and methods

Forty-four young canoeists were recruited for the study (mean ± SD, age: 15.11 ± 0.61 years; body height: 170.23 ± 8.63 cm; body mass: 62.81 ± 8.97 kg). The inclusion criteria were more than 5 years’ paddling experience and training at least six times per week. Paddlers were excluded if they presented pain induced or exacerbated by the test procedures, injury preventing participation in paddling training before testing or known structural spinal pathology.

### Procedures

An Institutional Ethical Committee approved the study and all subjects and parents or guardians signed an informed consent form before participation. The Spinal Mouse system (Idiag, Fehraltdorf, Switzerland), a hand-held, computer-assisted electromechanical-based device, was used to measure sagittal spinal curvatures and pelvic inclination in a relaxed standing position and in the boat. The measurements were made in a randomized order. No warm-up or stretching exercises were performed by the subjects prior to the test measurements. The subjects were allowed to rest briefly, standing up for 5 minutes between measurements. All measurements were made during the same testing session and were administered under the same environmental conditions. Participants were instructed not to undertake a weight-training session or strenuous exercise the day before testing to ensure consistent test conditions.

Prior to measurements, the principal researcher determined the spinous processus of C7 (starting point) and the top of the anal crease (end point) by palpation and marked these on the skin with a pencil. The Spinal Mouse was guided along the midline of the spine (or slightly paravertebrally in particularly thin individuals with prominent processus spinous) starting at the processus spinous of C7 and finishing at the top of the anal crease (approximately S3). For each testing position, the thoracic (T1–2 to T11–12) and lumbar (T12-L1 to the sacrum) spine and the pelvic inclination (difference between the sacral angle and the vertical) were recorded. In the lumbar curve, negative values corresponded to lumbar lordosis (posterior concavity). With respect to the pelvic inclination, a value of 0º represented the vertical position. Thus, a greater angle reflected an anterior pelvic tilt while a lower angle (negative values) reflected a posterior pelvic tilt.

### Measures

#### Standing

The subject assumed a relaxed position, with the head looking forward, the arms hanging by the side, the knees normally extended, and the feet shoulder-width apart.

#### Canoe positions

**Figure 1:**
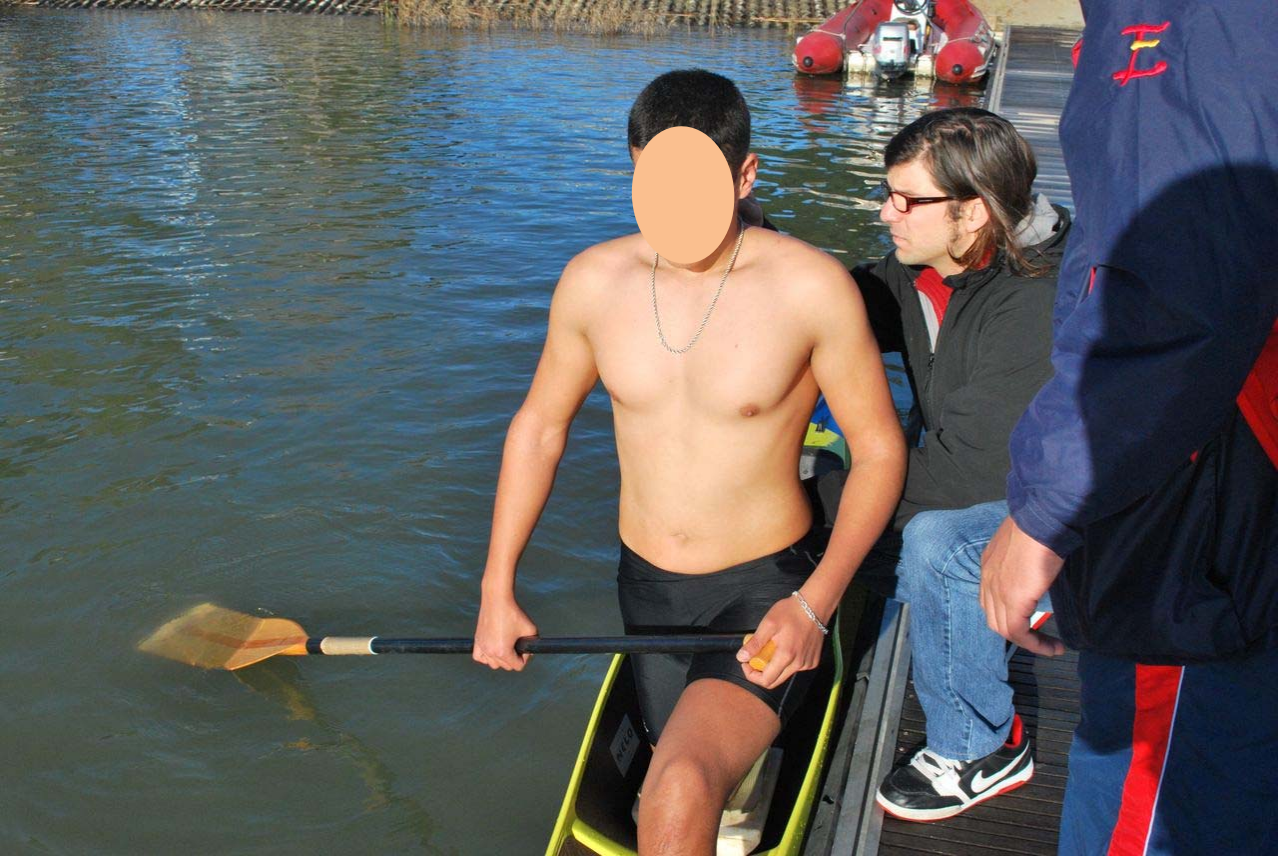
Base position in the canoe

**Figure 2:**
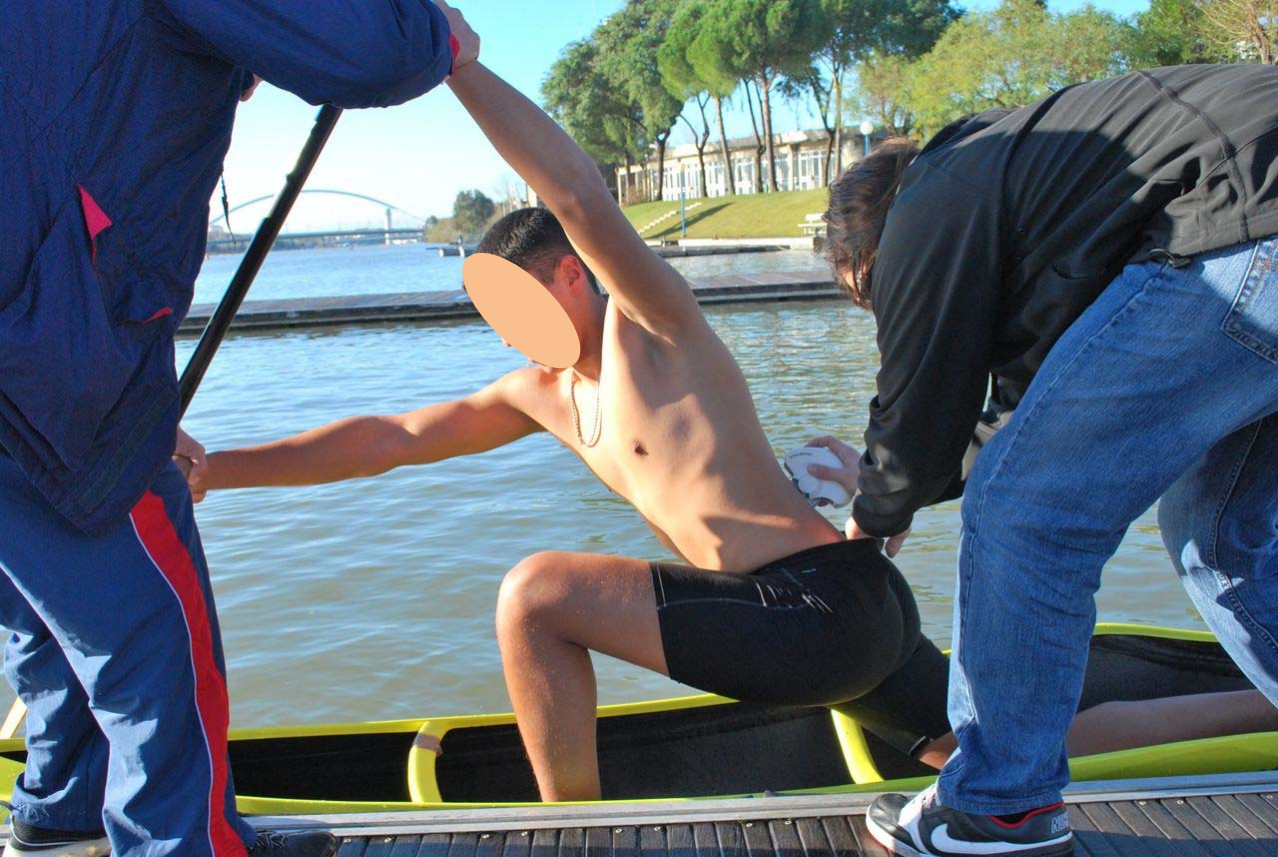
Catch phase position

Participants were measured under two different conditions:

- Base position. Canoeists were kneeling on one knee in their canoe (Figure 1).
- Catch phase of the stroke. Canoeists adopted the position at which the blade first contacts the water. When they reached this position, the canoeists were held at both hands by a researcher to maintain the posture (Figure 2).

In order to classify the posture in categories for thoracic kyphosis, the classification proposed by Mejia et al. (1996) was used: values between 20º and 45º were accepted as neutral thoracic kyphosis, values below 20º were considered to be thoracic hypokyphosis and values above 45º were considered to be thoracic hyperkyphosis. In lumbar curve, values between 20º and 40º were considered to be neutral, while values below 20º were considered to be hypolordotic and values above 40º were considered to be hyperlordotic (Tüzün et al., 1999).

### Statistical Analysis

The hypotheses of normality and homogeneity of the variance were analyzed via Kolmogorov-Smirnov and Levene tests, respectively. Descriptive statistics including means and standard deviations were calculated. One-way analysis of variance (ANOVA) with repeated measures was used to detect differences between the three positions evaluated in lumbar and thoracic curvatures and pelvic inclination. The significance of the multivariate repeated-measures analysis was confirmed by Wilk’s lambda, Pillai trace, Hotelling trace and Roy largest root tests, which yielded similar results. The sphericity assumption was tested using Mauchly’s test of sphericity. The Greenhouse-Geisser correction was applied if the assumption of sphericity was violated. If a significant *p*-value was identified for the main effect, pairwise comparisons were made using the Bonferroni correction for multiple comparisons with the significance criterion adjusted to 0.016. The data were analyzed using the SPSS 15.0. The level of significance was set at *p* ≤0.05.

## Results

**Figure 3:**
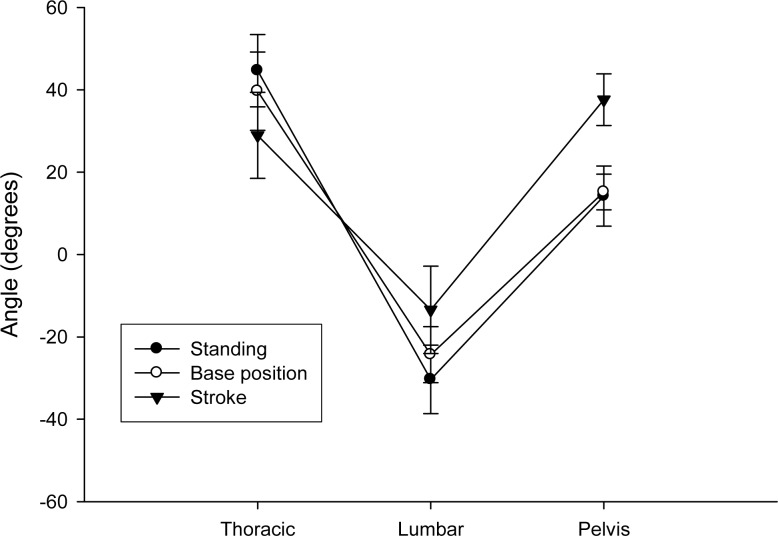
Mean (±SD) of the spinal curvatures and pelvic inclination while standing and in the boat

The mean values (± SD) of the thoracic and lumbar curves and pelvic inclination are presented in Figure 3. The ANOVA revealed significant differences for the main effects of thoracic and lumbar curves and pelvic inclination (*p*<0.001). Significant differences between all pairwise comparisons were found for thoracic kyphosis (*p*<0.001) and lumbar lordosis (*p*<0.001). With regard to pelvic position, significant differences were found between catch phase and the other two positions evaluated (*p*<0.001). However, no differences were found in pelvic inclination between a standing position and a base one.

The frequencies of each thoracic and lumbar curve categories in the three positions are presented in Table 1. A higher percentage of hyperkyphotic postures was found in standing than in the boat, while thoracic hypokyphosis increased in the catch phase. As long as lumbar curve was concerned, there was a tendency for lumbar flexion in the base position and the catch phase (Table 1).

## Discussion

The main objective of this study was to compare the spinal curvatures and pelvic position between standing and canoeing positions in young highly-trained canoeists. The main finding was the high frequency of thoracic hyperkyphotic posture in standing (43.2%) while the lumbar curve presented a high percentage of neutral postures (79.5%). These results are in accordance with previous studies in younger paddlers (13–14 years old) (López-Miñarro et al., 2008; López-Miñarro et al., 2010a,b; López-Miñarro and Alacid, 2010). However, in the canoe (in both base position and catch phase) the posture was characterized by reduced thoracic and lumbar curves.

Wojtys et al. (2000) reported that a high exposure of intensive athletic training might increase the risk of developing adolescent hyperkyphosis in certain sports. Förster et al. (2009) found an increased thoracic kyphosis and lumbar lordosis in climbers. In elite cross-country skiers, Alricsson and Werner (2006) found an increased thoracic kyphosis in skiers after a period of 5 years of intensive training. In a clinical screening of the Norwegian national team cross-country skiers, increased thoracic kyphosis were found in 66% of the skiers (Rusko et al., 2003). Rajabi et al. (2008) found that the thoracic kyphosis was higher in freestyle than in Greco-Roman wrestlers. These differences were related to the specific positions of each discipline. The freestyle routinely puts the spine in a more flexed position while the Greco-Roman style uses the spine in an almost erect position. Some studies have justified an increased thoracic kyphosis in relation to specific training postures (Alricsson and Werner, 2006; Rajabi et al., 2008), although these studies did not measure the specific posture during sport training.

The thoracic hyperkyphosis in the immature athlete could be related to excessive mechanical loading (Ashton-Miller, 2004). This increase might also be due to loss of disk height, which would tend to reduce the length of the anterior column of the spine, thereby increasing thoracic kyphosis (Wojtys et al., 2000). A possible reason for this could be long training with the spine in a static or cyclic flexed position. In the current study, we found a significant decrease of thoracic kyphosis in the base position (mean difference: 5.00º) and in the catch phase of the stroke (mean difference: 15.72º). In both positions, the cases of thoracic hyperkyphosis were more reduced. The position of upper limbs at the catch phase has been associated to thoracic and lumbar extension (Kapandji, 1980; Crosbie et al., 2008). Furthermore, the lower thoracic kyphosis may have been mediated by active support from muscle activation of spinal extensors and it may have beneficial effects for spinal loading. For these reasons, the increased thoracic kyphosis in standing might be more related to growth variables and spinal posture in the daily activities than to the specific position in the canoe. However, it is necessary to analyze the short-term effects of training in sagittal spinal curvatures and the spinal range of motion.

The spinal curvatures affect intradiscal pressures, compressive and shear forces in the intervertebral discs (Adams and Dolan, 1996; Wilke et al., 2001; Polga et al., 2004; Keller et al., 2008). Thoracic hyperkyphosis is associated with increased compression loading through the spine, which results in greater intervertebral disc loads (Keller et al., 2005) and low back pain (Harrison et al., 2005; Smith et al., 2008). Low back pain has been frequently reported in canoeists (Kameyama et al., 1993). Furthermore, increased thoracic kyphosis may bring the scapula into an anterior tilt and protracted position, so restricting subacromial space and the shoulder range of motion (Kebaetse et al., 1999; Finley and Lee, 2003).

The pelvis is considered as the base of the spine, and its anteroposterior orientation affects the sagittal curves of the spine (Levine and Whittle, 1996). The pelvic inclination was similar between standing and base position. At the catch phase of the stroke the pelvis anteriorly tilts to achieve a more efficient trunk-pelvic posture. Previous studies have found that pelvic inclination is conditioned by hamstring extensibility (Gajdosik et al., 1994; Carregaro and Coury, 2009; López-Miñarro and Alacid, 2010). Because the hamstring muscle originates on the ischial tuberosity of the pelvis, the tension in the hamstring has a direct influence on pelvic inclination during flexion movements, especially when knees are extended. However, in canoe paddling both knees are in flexed position but at the catch phase of the stroke, the pelvic inclination is greater. This position increases the tension in the hamstring muscles and gluteus maximus. An improved flexibility in both muscles could facilitate a more neutral posture in the lumbar spine.

The mean lumbar angle in standing and base position was neutral. The greater proportion of the canoeists showed neutral values in lumbar curve when standing. However, at the catch phase of the stroke, the mean value corresponded to lumbar hypolordosis. Lumbar lordosis is affected by the trunk-thigh angle. Hip flexion around 90° in one leg (base position) was associated with a reduced lumbar angle. However, no differences in pelvic position between standing and base position were detected. The frequency of neutral postures was more reduced at the catch phase because the lumbar spine performs an intervertebral flexion to increase the trunk inclination. In fact, some cases of lumbar kyphosis were detected in this position. No postures of lumbar kyphosis were found in standing or base position. A cyclic lumbar flexion for long periods has been related to cumulative trauma disorder (Lu et al., 2008) because it decreases the passive flexion stiffness of the lumbar spine due to viscoelastic creep (McGill and Brown, 1992) or stress-relaxation (Adams and Dolan, 1996) in the posterior lumbar tissues. This cyclic position might lead to the development of a decreased lumbar curvature over time in growing individuals. However, the greater percentage of canoeists had neutral angles in standing. In agreement with previous studies on younger paddlers (López-Miñarro and Alacid, 2010; López-Miñarro et al., 2010a,b; López-Miñarro et al., 2008), only a few canoeists showed hypolordotic postures in standing.

The traditional training of paddlers usually does not include postural activities of the spine and pelvis. Postural screening programs and postural retraining in paddlers with poor posture may be relevant. Postural activities should be incorporated into the training activities of canoeists to improve the thoracic posture in standing and to reduce the lumbar kyphotic postures at the catch phase of the stroke. Care should be taken to ensure technically correct performance of the sport movements and correct spinal postures in the boat.

With regards to practical implications, systematic hamstring stretching and trunk strength exercises should be included in the training program of sport disciplines where a significant forward lean occurs in training and competition. In all stretching exercises the subjects should flex their hip or trunk maintaining their spine as aligned as possible (for example, unilateral supine hamstring stretch and unilateral standing stretch). Seated stretch is only recommended if the subjects have sufficient hamstring extensibility to ensure an upright torso and neutral posture (McGill, 2002). Lateral and abdominal muscles (quadratus lumborum, abdominal obliques and transversus abdominis) are important for optimal stability, and are targeted with the prone bridge, isometric side support and curl-ups in stable and unstable conditions. Furthermore, lumbar exercises such us bird-dog, trunk extension and supine bridge are recommended. The goal is to ensure a stable spine.

In conclusion, the highly-trained young canoeists showed a greater frequency of standing thoracic hyperkyphosis, which may be related to factors other than the posture and movement in the canoe. The canoeists adopt a more flexed posture in the lumbar spine at the catch phase of the stroke, although this position may not affect the sagittal configuration in standing. Postural training should be included in the training programs to improve the thoracic posture in standing and reduce the lumbar flexion at the catch phase of the stroke.

## Figures and Tables

**Table 1:** Percentage of canoeists in each category of thoracic kyphosis and lumbar lordosis in three positions

	**Thoracic kyphosis**		

	Standing	Base position	Catch phase
	
Hypokyphosis (<20º)	0%	0%	18,2%
Neutral (20–45º)	56,8%	70,5%	75%
Hyperkyphosis (>45º)	43,2%	29,5%	6,8%

	**Lumbar lordosis**		

	Standing	Base position	Catch phase
	
Lumbar inversion (>1º)	0%	0%	13,6%
Hypolordosis (0 / < −20º)	9,1%	20,5%	59,1%
Neutral (−20 / −40º)	79,5%	79,5%	27,3%
Hyperlordosis (>−40º)	11,4%	0%	0%
